# 1,4-Diazo­niabicyclo­[2.2.2]octane terephthalate

**DOI:** 10.1107/S1600536808025312

**Published:** 2008-08-16

**Authors:** E Yang, Xu-Chun Song, Jian-Wei Zhu

**Affiliations:** a College of Chemistry and Materials Science, Fujian Normal University, Fuzhou 350007, People’s Republic of China; b College of Chemistry and Chemical Engineering, Xiamen University, Xiamen 361005, People’s Republic of China

## Abstract

In the title compound, C_6_H_14_N^2+^·C_8_H_4_O_4_^2−^, the protonated 1,4-diazo­niabicyclo­[2.2.2]octane cations and the deprotonated terephthalate anions are alternately linked by N—H⋯O hydrogen bonds into chains.
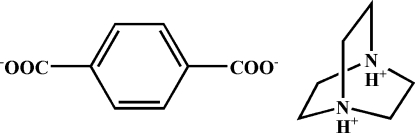

## Experimental

### 

#### Crystal data

C_6_H_14_N_2_^2+^·C_8_H_4_O_4_^2−^
*M* *_r_* = 278.30
Triclinic, 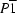

*a* = 7.8046 (10) Å
*b* = 9.5482 (2) Å
*c* = 10.8075 (2) Å
α = 65.900 (10)°
β = 78.360 (10)°
γ = 66.800 (10)°
*V* = 672.39 (2) Å^3^
*Z* = 2
Mo *K*α radiation
μ = 0.10 mm^−1^
*T* = 293 (2) K
0.23 × 0.13 × 0.08 mm

#### Data collection

Siemens SMART 1K CCD area-detector diffractometer
Absorption correction: multi-scan (*SADABS*; Sheldrick, 1996 ▶) *T*_min_ = 0.900, *T*_max_ = 0.950
7312 measured reflections
2377 independent reflections
1779 reflections with *I* > 2σ(*I*)
*R*_int_ = 0.026

#### Refinement

*R*[*F* ^2^ > 2σ(*F* ^2^)] = 0.043
*wR*(*F* ^2^) = 0.125
*S* = 1.04
2377 reflections
188 parameters
2 restraints
H atoms treated by a mixture of independent and constrained refinement
Δρ_max_ = 0.21 e Å^−3^
Δρ_min_ = −0.27 e Å^−3^

### 

Data collection: *SMART* (Siemens, 1996 ▶); cell refinement: *SAINT* (Siemens, 1996 ▶); data reduction: *SAINT*; program(s) used to solve structure: *SHELXS97* (Sheldrick, 2008 ▶); program(s) used to refine structure: *SHELXL97* (Sheldrick, 2008 ▶); molecular graphics: *DIAMOND* (Bergerhoff *et al.*, 1996 ▶); software used to prepare material for publication: *SHELXTL* (Sheldrick, 2008 ▶).

## Supplementary Material

Crystal structure: contains datablocks I, global. DOI: 10.1107/S1600536808025312/pv2092sup1.cif

Structure factors: contains datablocks I. DOI: 10.1107/S1600536808025312/pv2092Isup2.hkl

Additional supplementary materials:  crystallographic information; 3D view; checkCIF report

## Figures and Tables

**Table 1:** Hydrogen-bond geometry (Å, °)

*D*—H⋯*A*	*D*—H	H⋯*A*	*D*⋯*A*	*D*—H⋯*A*
N1—H1*N*⋯O2	0.954 (15)	1.623 (15)	2.5757 (19)	176.8 (17)
N2—H2*N*⋯O4^i^	0.959 (15)	1.600 (15)	2.5589 (19)	177.9 (18)

Symmetry code: (i) 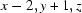.

**Fig. 1.**
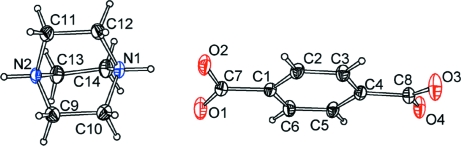
A view of the title compound, showing 30% probability displacement ellipsoids.

**Fig. 2.**
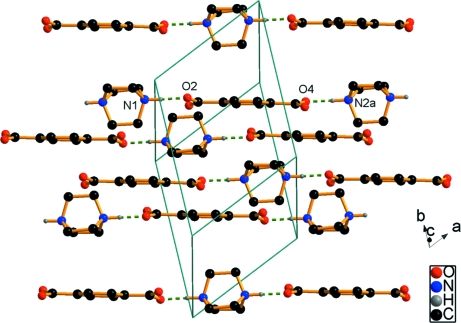
The unit-cell packing of the title compound, showing the hydrogen bonding chains (symmetry code: a = 2 + x, -1 + y, z).

## supplementary crystallographic information

### Comment

The asymmetric unit of the title compound, (I), consists of a protonated 1,4-diazoniabicyclo[2.2.2]octane cation, C_6_H_14_N^2+^, and a deprotonated terephthalate anion, C_8_H_4_O_4_^2-^, (Figure 1). Single N—H···O hydrogen bond was formed between the protonated N end of the cation and the deprotonated carboxylate group of the anion, which generates the hydrogen bonding chains (Table 1 & Figure 2).

### Experimental

A mixture of 1,4-diazoniabicyclo[2.2.2]octane (0.072 g), terephthalic acid (0.08 g) and H_2_O (10 ml) was sealed in a 25 ml stainless-steel reactor with a Teflon-lined stainless steel reactor and was heated at 373 K for 3 d. On completion of the reaction, the reactor was cooled slowly to room temperature and the mixture was filtered, giving collorless single crystals suitable for X-ray analysis.

### Refinement

The H atoms bonded to C atoms were placed at calculated positions at C—H distances 0.93 and 0.97 Å for the aryl and methylene H-atoms, respectively, and *U*_iso_(H) = 1.2*U*_eq_(C) using a riding model. The H atoms bonded to N atoms were refined freely.

### Crystal data

**Table:**

C_6_H_14_N_2_^2+^·C_8_H_4_O_4_^2–^	*Z* = 2
*M**_r_* = 278.30	*F*_000_ = 296
Triclinic, *P*1	*D*_x_ = 1.375 Mg m^−^^3^
Hall symbol: -P 1	Mo *K*α radiation λ = 0.71073 Å
*a* = 7.8046 (10) Å	Cell parameters from 98 reflections
*b* = 9.5482 (2) Å	θ = 2.1–25.0º
*c* = 10.8075 (2) Å	µ = 0.10 mm^−^^1^
α = 65.990 (10)º	*T* = 293 (2) K
β = 78.436 (10)º	Prism, colorless
γ = 66.180 (10)º	0.23 × 0.13 × 0.08 mm
*V* = 672.39 (2) Å^3^	

### Data collection

**Table:**

Siemens SMART 1K CCD area-detector diffractometer	2377 independent reflections
Radiation source: fine-focus sealed tube	1779 reflections with *I* > 2σ(*I*)
Monochromator: graphite	*R*_int_ = 0.026
*T* = 293(2) K	θ_max_ = 25.0º
φ and ω scans	θ_min_ = 2.1º
Absorption correction: multi-scan(SADABS; Sheldrick, 1996)	*h* = −9→9
*T*_min_ = 0.900, *T*_max_ = 0.950	*k* = −11→11
7312 measured reflections	*l* = −12→12

### Refinement

**Table:**

Refinement on *F*^2^	Secondary atom site location: difference Fourier map
Least-squares matrix: full	Hydrogen site location: inferred from neighbouring sites
*R*[*F*^2^ > 2σ(*F*^2^)] = 0.043	H atoms treated by a mixture of independent and constrained refinement
*wR*(*F*^2^) = 0.125	*w* = 1/[σ^2^(*F*_o_^2^) + (0.0633*P*)^2^ + 0.1475*P*] where *P* = (*F*_o_^2^ + 2*F*_c_^2^)/3
*S* = 1.04	(Δ/σ)_max_ = 0.002
2377 reflections	Δρ_max_ = 0.21 e Å^−^^3^
188 parameters	Δρ_min_ = −0.26 e Å^−^^3^
2 restraints	Extinction correction: SHELXL97 (Sheldrick, 2008), Fc^*^=kFc[1+0.001xFc^2^λ^3^/sin(2θ)]^-1/4^
Primary atom site location: structure-invariant direct methods	Extinction coefficient: 0.023 (4)

### Special details

**Table:**

Geometry. All e.s.d.'s (except the e.s.d. in the dihedral angle between two l.s. planes) are estimated using the full covariance matrix. The cell e.s.d.'s are taken into account individually in the estimation of e.s.d.'s in distances, angles and torsion angles; correlations between e.s.d.'s in cell parameters are only used when they are defined by crystal symmetry. An approximate (isotropic) treatment of cell e.s.d.'s is used for estimating e.s.d.'s involving l.s. planes.
Refinement. Refinement of *F*^2^ against ALL reflections. The weighted *R*-factor *wR* and goodness of fit *S* are based on *F*^2^, conventional *R*-factors *R* are based on *F*, with *F* set to zero for negative *F*^2^. The threshold expression of *F*^2^ > σ(*F*^2^) is used only for calculating *R*-factors(gt) *etc*. and is not relevant to the choice of reflections for refinement. *R*-factors based on *F*^2^ are statistically about twice as large as those based on *F*, and *R*- factors based on ALL data will be even larger.

### Fractional atomic coordinates and isotropic or equivalent isotropic displacement parameters (Å^2^)

**Table:**

	*x*	*y*	*z*	*U*_iso_*/*U*_eq_	
O1	0.2883 (2)	0.8953 (2)	0.60534 (16)	0.0691 (5)	
O2	0.25555 (18)	0.84559 (17)	0.82616 (14)	0.0489 (4)	
O3	1.1805 (2)	0.27977 (19)	0.89737 (15)	0.0629 (5)	
O4	1.23265 (18)	0.40326 (18)	0.67751 (14)	0.0519 (4)	
N1	−0.0871 (2)	1.03609 (18)	0.77218 (15)	0.0328 (4)	
H1N	0.040 (2)	0.964 (2)	0.7898 (18)	0.039*	
N2	−0.4239 (2)	1.21700 (18)	0.72154 (14)	0.0317 (4)	
H2N	−0.553 (2)	1.285 (2)	0.7069 (18)	0.038*	
C1	0.5526 (2)	0.7124 (2)	0.73811 (18)	0.0315 (4)	
C2	0.6233 (2)	0.6175 (2)	0.86638 (18)	0.0345 (4)	
H2A	0.5455	0.6256	0.9425	0.041*	
C3	0.8080 (3)	0.5111 (2)	0.88253 (18)	0.0354 (4)	
H3A	0.8524	0.4464	0.9692	0.042*	
C4	0.9270 (2)	0.5006 (2)	0.77020 (17)	0.0303 (4)	
C5	0.8571 (3)	0.5980 (2)	0.64181 (18)	0.0352 (4)	
H5A	0.9362	0.5932	0.5657	0.042*	
C6	0.6722 (3)	0.7017 (2)	0.62577 (19)	0.0376 (5)	
H6A	0.6273	0.7649	0.5391	0.045*	
C7	0.3509 (3)	0.8265 (2)	0.71843 (19)	0.0376 (5)	
C8	1.1275 (3)	0.3846 (2)	0.78670 (19)	0.0356 (4)	
C9	−0.3943 (3)	1.0480 (2)	0.7369 (2)	0.0389 (5)	
H9A	−0.4315	1.0493	0.6559	0.047*	
H9B	−0.4709	1.0034	0.8132	0.047*	
C10	−0.1869 (3)	0.9399 (2)	0.7601 (2)	0.0407 (5)	
H10A	−0.1752	0.8438	0.8422	0.049*	
H10B	−0.1322	0.9032	0.6847	0.049*	
C11	−0.3778 (3)	1.2165 (3)	0.84800 (19)	0.0419 (5)	
H11A	−0.4643	1.1827	0.9213	0.050*	
H11B	−0.3897	1.3264	0.8360	0.050*	
C12	−0.1769 (3)	1.0977 (2)	0.88353 (18)	0.0377 (5)	
H12A	−0.1062	1.1538	0.8965	0.045*	
H12B	−0.1781	1.0064	0.9672	0.045*	
C13	−0.3010 (3)	1.2820 (2)	0.60835 (19)	0.0404 (5)	
H13A	−0.3256	1.3954	0.5939	0.048*	
H13B	−0.3262	1.2783	0.5256	0.048*	
C14	−0.0959 (3)	1.1773 (2)	0.64345 (19)	0.0411 (5)	
H14A	−0.0299	1.1377	0.5711	0.049*	
H14B	−0.0356	1.2435	0.6526	0.049*	

### Atomic displacement parameters (Å^2^)

**Table:**

	*U*^11^	*U*^22^	*U*^33^	*U*^12^	*U*^13^	*U*^23^
O1	0.0381 (9)	0.0919 (13)	0.0523 (10)	0.0164 (8)	−0.0141 (7)	−0.0364 (9)
O2	0.0265 (7)	0.0596 (10)	0.0459 (8)	0.0020 (7)	0.0000 (6)	−0.0230 (7)
O3	0.0341 (8)	0.0626 (10)	0.0484 (9)	0.0015 (7)	−0.0034 (7)	0.0032 (8)
O4	0.0259 (7)	0.0630 (10)	0.0413 (8)	0.0009 (7)	0.0016 (6)	−0.0126 (7)
N1	0.0195 (8)	0.0335 (9)	0.0379 (9)	−0.0030 (7)	−0.0017 (6)	−0.0122 (7)
N2	0.0203 (8)	0.0342 (9)	0.0353 (8)	−0.0016 (7)	−0.0034 (6)	−0.0145 (7)
C1	0.0242 (9)	0.0320 (10)	0.0394 (10)	−0.0070 (8)	−0.0002 (8)	−0.0177 (8)
C2	0.0277 (10)	0.0387 (11)	0.0348 (10)	−0.0085 (8)	0.0063 (8)	−0.0181 (8)
C3	0.0316 (10)	0.0363 (10)	0.0305 (9)	−0.0076 (8)	−0.0022 (8)	−0.0091 (8)
C4	0.0248 (9)	0.0272 (9)	0.0360 (10)	−0.0076 (8)	0.0007 (8)	−0.0116 (8)
C5	0.0270 (10)	0.0388 (11)	0.0329 (10)	−0.0059 (8)	0.0043 (8)	−0.0149 (8)
C6	0.0297 (10)	0.0403 (11)	0.0325 (10)	−0.0027 (8)	−0.0048 (8)	−0.0115 (8)
C7	0.0270 (10)	0.0403 (11)	0.0456 (11)	−0.0045 (8)	−0.0033 (9)	−0.0226 (9)
C8	0.0263 (10)	0.0344 (10)	0.0399 (11)	−0.0069 (8)	−0.0030 (8)	−0.0108 (9)
C9	0.0333 (11)	0.0375 (11)	0.0475 (11)	−0.0130 (9)	−0.0045 (9)	−0.0156 (9)
C10	0.0378 (11)	0.0316 (10)	0.0518 (12)	−0.0081 (9)	−0.0050 (9)	−0.0173 (9)
C11	0.0308 (11)	0.0532 (13)	0.0422 (11)	−0.0041 (9)	−0.0019 (8)	−0.0285 (10)
C12	0.0311 (10)	0.0438 (11)	0.0365 (10)	−0.0076 (9)	−0.0059 (8)	−0.0170 (9)
C13	0.0360 (11)	0.0348 (11)	0.0397 (10)	−0.0087 (9)	−0.0027 (8)	−0.0068 (9)
C14	0.0303 (10)	0.0420 (11)	0.0414 (11)	−0.0105 (9)	0.0028 (8)	−0.0104 (9)

### Geometric parameters (Å, °)

**Table:**

O1—C7	1.221 (2)	C4—C8	1.503 (2)
O2—C7	1.288 (2)	C5—C6	1.378 (3)
O3—C8	1.222 (2)	C5—H5A	0.9300
O4—C8	1.287 (2)	C6—H6A	0.9300
N1—C10	1.478 (2)	C9—C10	1.533 (3)
N1—C12	1.480 (2)	C9—H9A	0.9700
N1—C14	1.482 (2)	C9—H9B	0.9700
N1—H1N	0.954 (15)	C10—H10A	0.9700
N2—C9	1.477 (2)	C10—H10B	0.9700
N2—C11	1.479 (2)	C11—C12	1.531 (3)
N2—C13	1.481 (2)	C11—H11A	0.9700
N2—H2N	0.959 (15)	C11—H11B	0.9700
C1—C6	1.388 (2)	C12—H12A	0.9700
C1—C2	1.388 (3)	C12—H12B	0.9700
C1—C7	1.507 (2)	C13—C14	1.532 (3)
C2—C3	1.383 (3)	C13—H13A	0.9700
C2—H2A	0.9300	C13—H13B	0.9700
C3—C4	1.385 (2)	C14—H14A	0.9700
C3—H3A	0.9300	C14—H14B	0.9700
C4—C5	1.390 (2)		
			
C10—N1—C12	109.46 (14)	N2—C9—C10	109.63 (14)
C10—N1—C14	109.60 (14)	N2—C9—H9A	109.7
C12—N1—C14	109.29 (14)	C10—C9—H9A	109.7
C10—N1—H1N	106.8 (11)	N2—C9—H9B	109.7
C12—N1—H1N	111.5 (11)	C10—C9—H9B	109.7
C14—N1—H1N	110.1 (11)	H9A—C9—H9B	108.2
C9—N2—C11	109.82 (14)	N1—C10—C9	109.09 (14)
C9—N2—C13	109.48 (14)	N1—C10—H10A	109.9
C11—N2—C13	109.17 (15)	C9—C10—H10A	109.9
C9—N2—H2N	109.9 (11)	N1—C10—H10B	109.9
C11—N2—H2N	106.9 (11)	C9—C10—H10B	109.9
C13—N2—H2N	111.5 (11)	H10A—C10—H10B	108.3
C6—C1—C2	118.69 (16)	N2—C11—C12	109.40 (14)
C6—C1—C7	119.60 (16)	N2—C11—H11A	109.8
C2—C1—C7	121.71 (16)	C12—C11—H11A	109.8
C3—C2—C1	120.90 (16)	N2—C11—H11B	109.8
C3—C2—H2A	119.6	C12—C11—H11B	109.8
C1—C2—H2A	119.6	H11A—C11—H11B	108.2
C2—C3—C4	120.26 (17)	N1—C12—C11	109.31 (14)
C2—C3—H3A	119.9	N1—C12—H12A	109.8
C4—C3—H3A	119.9	C11—C12—H12A	109.8
C3—C4—C5	118.80 (16)	N1—C12—H12B	109.8
C3—C4—C8	120.66 (16)	C11—C12—H12B	109.8
C5—C4—C8	120.54 (16)	H12A—C12—H12B	108.3
C6—C5—C4	120.92 (16)	N2—C13—C14	108.99 (15)
C6—C5—H5A	119.5	N2—C13—H13A	109.9
C4—C5—H5A	119.5	C14—C13—H13A	109.9
C5—C6—C1	120.40 (17)	N2—C13—H13B	109.9
C5—C6—H6A	119.8	C14—C13—H13B	109.9
C1—C6—H6A	119.8	H13A—C13—H13B	108.3
O1—C7—O2	124.21 (17)	N1—C14—C13	109.66 (14)
O1—C7—C1	120.01 (17)	N1—C14—H14A	109.7
O2—C7—C1	115.77 (16)	C13—C14—H14A	109.7
O3—C8—O4	124.30 (17)	N1—C14—H14B	109.7
O3—C8—C4	120.41 (17)	C13—C14—H14B	109.7
O4—C8—C4	115.28 (16)	H14A—C14—H14B	108.2

### Hydrogen-bond geometry (Å, °)

**Table:**

*D*—H···*A*	*D*—H	H···*A*	*D*···*A*	*D*—H···*A*
N1—H1N···O2	0.954 (15)	1.623 (15)	2.5757 (19)	176.8 (17)
N2—H2N···O4^i^	0.959 (15)	1.600 (15)	2.5589 (19)	177.9 (18)

Symmetry codes: (i) *x*−2, *y*+1, *z*.
